# TDP-43 proteinopathy as a biomarker and therapeutic target in amyotrophic lateral sclerosis

**DOI:** 10.1042/BST20260896

**Published:** 2026-07-01

**Authors:** Eleni Christoforidou, Emily McFagan, Martha McLaughlin, Majid Hafezparast

**Affiliations:** Department of Neuroscience, School of Life Sciences, University of Sussex, Brighton, U.K.

**Keywords:** ALS, Amyotrophic Lateral Sclerosis, MND, Motor Neuron Disease, TARDBP, TDP-43

## Abstract

Amyotrophic lateral sclerosis (ALS) is the most common form of adult-onset motor neuron disease, characterised by the degeneration of upper and lower motor neurons. The cytoplasmic aggregation of TDP-43 (TAR DNA-binding protein 43), an RNA-binding protein, is considered a hallmark of ALS pathology, found in nearly all postmortem cases of ALS. TDP-43 is normally primarily nuclear, where it has a widespread role in gene regulation. Mutations, extrinsic stressors, and alterations in RNA homeostasis in ALS lead to nuclear depletion of TDP-43 and the formation of cytosolic TDP-43 aggregates. This causes multiple downstream effects on neuronal function and degeneration as well as gene expression. TDP-43 is a promising target as a biomarker, as it is found to be elevated in the biofluids of ALS patients, and its cytoplasmic aggregation can also be observed in peripheral tissues; however, methodological variability and technical limitations currently preclude the establishment of TDP-43 as a standalone biomarker. There are also promising therapeutic strategies in development targeting TDP-43 pathology, but a critical challenge that remains is achieving a balance between eliminating toxic aggregates and preserving the essential functions of TDP-43. In summary, with further research, considering TDP-43 pathology in ALS gives hope for finding future novel diagnostics and therapeutics for ALS.

## Introduction

Amyotrophic lateral sclerosis (ALS) has an incidence of approximately 2 in 100,000 people [1], and symptoms include skeletal muscle weakness and paralysis that usually begin in late middle age and result in fatality 2–5 years after diagnosis [2]. Most cases are sporadic, and no strongly correlating environmental risk factors have been identified [3,4]. Genetic causes can be identified for 11% of sporadic and 68% of familial cases with mutations in over 50 genes associated with the disease [5]. Despite the heterogeneity of ALS, evidence implicates the RNA-binding protein TDP-43 (TAR DNA-binding protein 43) as a major disease protein. Neuronal TDP-43 cytoplasmic inclusions are found in 97% of all ALS patients [6,7], and over 50 ALS-associated *TARDBP* mutations have been identified [8–11], conferring both gain and loss of function of TDP-43 [7]. Here, we review the pathological function of TDP-43 in ALS and the recent innovations and challenges in developing ALS biomarkers and therapeutics targeting TDP-43 pathology. Finally, this review extends existing discussions of TDP-43 pathology in ALS by integrating recent findings from physiologically relevant disease models and non-neuronal cell populations, as well as advances in the potential of pathological TDP-43 as a diagnostic tool in ALS, areas that remain comparatively underrepresented in prior reviews.

## Structure, localisation, and function of TDP-43

**Figure 1:**
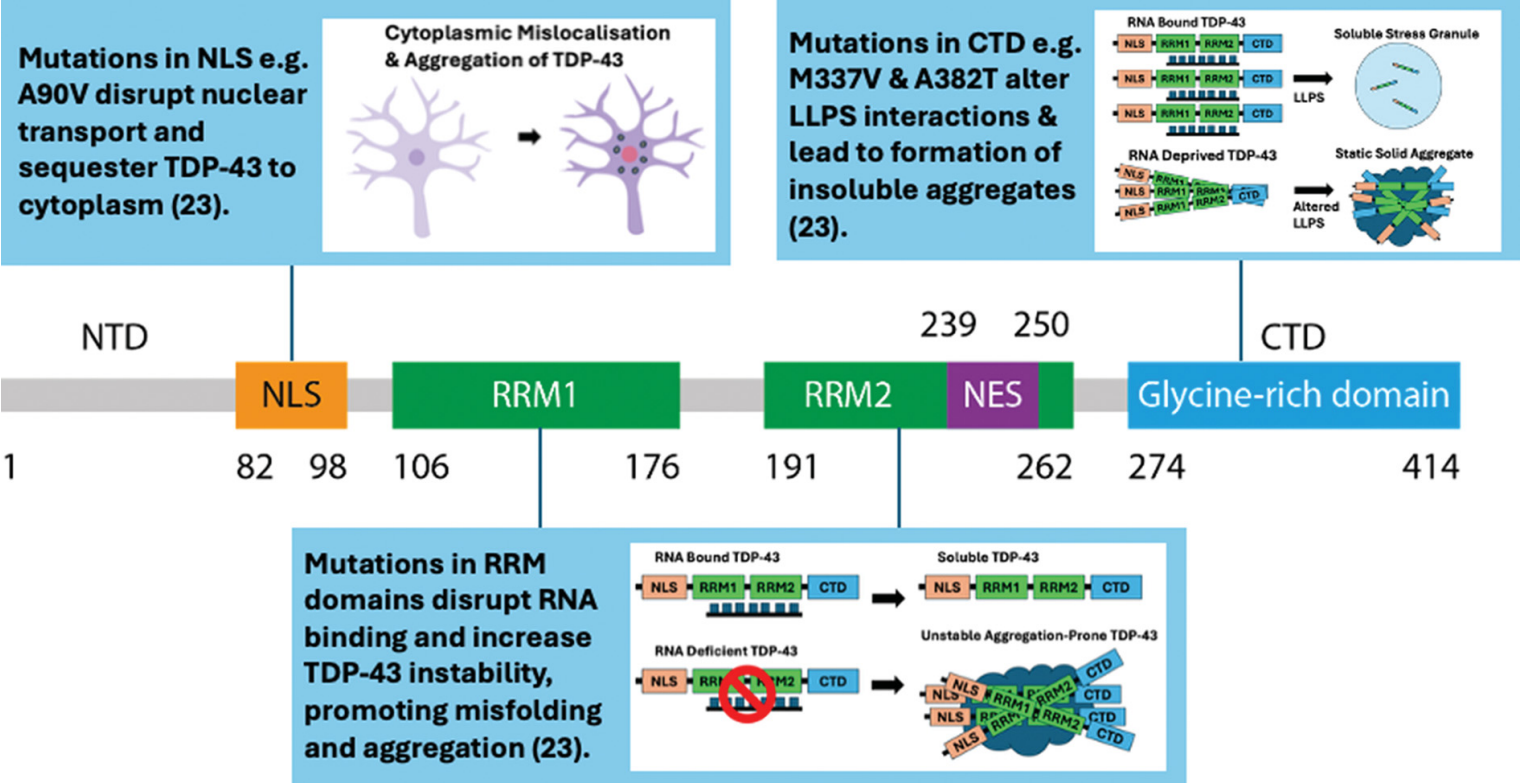
Schematic representation of the structure of the TDP-43 protein The figure shows the four domains of the protein, with the numbers corresponding to the amino acid positions of the beginning and end of each domain. The NTD contains a fold involved in the formation of dimers or oligomers [18,19]. RNA recognition motifs RRM1 and RRM2 are necessary for binding to DNA and RNA molecules [20], and the CTD plays a role in protein-protein interactions [21]. The nuclear localisation signal (NLS) and nuclear export signal (NES) allow for nucleocytoplasmic shuttling of TDP-43 [22]. Schematics represent how mutations in each region contribute to TDP-43 pathology (reviewed in [23]). Figure adapted from [24].

The *TARDBP* gene is located on chromosome 1 in humans and is translated into the 414 amino acid-long TDP-43. The protein is made up of four domains (Figure 1): the N-terminal domain (NTD), two folded RNA recognition motifs (RRM1 and RRM2), and the C-terminal domain (CTD) containing a glycine-rich region. TDP-43 is typically localised to the nucleus and expressed in almost all tissues, with its expression tightly controlled through TDP-43 binding to the 3′ untranslated region (UTR) of its own mRNA transcript, creating an autoregulatory feedback loop [12–16]. In neurons, TDP-43 is transported bidirectionally along axons and dendrites in granules that associate with mRNA transport proteins and RNA [17].

TDP-43 is a DNA/RNA-binding protein and plays a multifaceted role in gene regulation, involved in transcriptional repression and mRNA translation, stability, and splicing [24]. It preferentially binds to ‘UG’-rich dinucleotide repeats, and most pre-RNA binding sites for TDP-43 are in intronic regions [25,26]. TDP-43 binds to many neuronal RNAs, particularly those involved in neuronal development, RNA metabolism, and synaptic function. TDP-43 also binds to the pre-mRNAs of the ALS risk genes *STMN2* and *UNC13A* [27–29] as well as RNAs encoding the neurodegeneration-linked proteins FUS, tau, progranulin, and ataxins 1 and 2 [30]. Complete knockout of TDP-43 in mice causes embryonic lethality [31], highlighting its essential role. TDP-43 is also important for motor neuron survival; conditional knockout in mouse motor neurons leads to reduced body weight, muscle atrophy, and motor neuron loss, with evidence of impaired autophagy marked by decreased ATG7 levels and accumulation of p62 and ubiquitin-positive inclusions [32].

## Mechanisms and impacts of TDP-43 dysfunction in ALS

### Mechanisms

Cytoplasmic aggregation of TDP-43 is important to the pathogenesis of ALS and is driven by a complex interplay between the intrinsic propensity of TDP-43 to self-associate and undergo liquid–liquid phase separation (LLPS) and its RNA-binding functions. Binding to RNA retains TDP-43 in a soluble, functional state [33,34], whilst selective binding to UG-rich RNA sequences via its RRM domains [26,35–37] modulates its folding state and phase behaviour [34,38]. When the RNA binding of TDP-43 is compromised, due either to mutations within the RRM regions (Figure 1) [39,40] or through post-translational modifications, the protective effects of RNA are lost, leading to increased TDP-43 instability and a heightened propensity for misfolding and aggregation [41,42]. TDP-43 also undergoes LLPS through the stress granule (SG)-dependent pathway, a reversible process by which it forms dynamic, liquid-like condensates, triggered by the recruitment of TDP-43 into SGs under cellular stress [43–46]. Chronic stress can lead to the persistence of TDP-43-containing SGs that gradually transition into static, solid aggregates, marked by features such as hyperphosphorylation [47–49]. Alternatively, SG-independent LLPS involves the formation of RNA-deficient droplets driven largely by interactions within the intrinsically disordered C-terminal low-complexity domain of TDP-43, in which most ALS-associated mutations are contained [6,33,34,50,51]. Post-translational modifications also play a significant role in modulating TDP-43 aggregation. For example, phosphorylation at residues such as serines 409/410 is a hallmark of disease and is found predominantly in the detergent-insoluble fractions of ALS tissues [6,52]. Similarly, acetylation of key lysine residues impairs RNA binding and enhances the aggregation propensity of TDP-43 [53–55].

Mislocalisation of TDP-43 from the nucleus to the cytoplasm is another central event underlying its pathogenicity. Under normal conditions, TDP-43 shuttles between the nucleus and cytoplasm to carry out RNA processing functions [56–58]. However, defects in nucleocytoplasmic transport, often linked to *C9ORF72* hexanucleotide repeat expansions; disruptions in nuclear pore complex components [43,59]; and impaired TDP-43 dimerization [60] can lead to cytoplasmic accumulation of TDP-43. This not only deprives the nucleus of the normal functions of TDP-43 but also promotes toxic gain-of-function effects in the cytoplasm. In the cytoplasm, TDP-43 interacts aberrantly with other proteins and organelles, contributing to mitochondrial dysfunction, oxidative stress, and the disruption of intracellular signalling pathways [44,61,62]. Mislocalisation is also highly interwined with TDP-43 aggregation. In the cytoplasm, where RNA concentrations are lower than in the nucleus, TDP-43 is more prone to undergo irreversible phase transition into solid aggregates that are unable to be cleared by the ubiquitin-proteasome system (UPS) [63,64]. Cytoplasmic aggregates also increasingly sequester physiological TDP-43 from the nucleus [65], damage the nuclear pore complex, and disrupt nucleocytoplasmic shuttling of nuclear proteins and mRNA [66].

Pathological hallmarks of TDP-43 are also largely conserved across hereditary and sporadic ALS [23], despite heterogeneous upstream triggers. Mutations in *TARDBP* directly promote TDP-43 misfolding and disrupt RNA metabolism, while other mutations act indirectly through impaired nucleocytoplasmic transport and proteostasis (Figure 1) [43,59,67–69]. As the onset of sporadic ALS is predominantly in middle-to-late life, it is thought to arise from multi-step [70], potentially oligogenic [71], age-associated cellular decline. In post-mortem samples of individuals aged over 65, TDP-43 proteinopathy is observed even in the absence of a diagnosis of ALS or frontotemporal dementia (FTD) [72]. This may be downstream of proteostasis failure, impaired autophagy and lysosomal degradation, chronic cellular stress, or axonal transport deficits [73–75]. These processes are interdependent and may form self-reinforcing feedback loops that can cause a transition into pathological states. Deciphering these events will help elucidate causative mechanisms of the multifactorial onset of TDP-43 proteinopathy in sporadic ALS.

### Impacts

Cytoplasmic inclusions of TDP-43 can cause toxicity directly, contributing to disease pathology. Aggregated TDP-43 protein is considered to have prion-like properties, capable of propagating between cells and potentially playing a key role in the progression of disease (reviewed in [76]). Briefly, TDP-43 aggregation disrupts autoregulation, leading to TDP-43 overexpression, which has previously been found to be toxic *in vivo* and *in vitro* [77], and can also trigger the aggregation of other pathogenic proteins, impacting many cellular pathways (reviewed in [78]). Cytoplasmic TDP-43 may also impair the activity of the UPS, impacting the cellular capacity to clear misfolded proteins, promoting further aggregation [79].

The loss of TDP-43 nuclear function has been shown to have wide-ranging effects, reflecting its multifaceted role in gene regulation; however, of recent interest is the inclusion of cryptic exons [80], non-conserved, canonically intronic sequences that are aberrantly included in mature RNA, producing novel RNA isoforms that are degraded through nonsense-mediated decay (NMD) or by the production of a cryptic peptide. Notably two ALS genes, Stathmin 2 (*STMN2*), which has a role in microtubule stability and axonal outgrowth, and *UNC13A*, which has a role in neurotransmitter release and synaptic transmission, are prominent targets. TDP-43 knockdown in human motor neurons derived from induced pluripotent stem cells (iPSCs) results in decreased levels of STMN2 [81]. Down-regulation of TDP-43 in human iPSC-derived cortical neurons via CRISPR inhibition results in the inclusion of cryptic exons in *UNC13A*, leading to protein loss through NMD [29,82–84]. Interestingly, the inclusion of this cryptic exon in *UNC13A* is enhanced by single nuclear polymorphisms that have been shown to increase risk and negatively affect survival time in ALS.

Alternative polyadenylation has also recently emerged as a core function of TDP-43 [85]. Absence of nuclear TDP-43 results in altered 3′ UTR regions in over 3000 mRNA transcripts. This is primarily through 3′ UTR lengthening and premature polyadenylation, which compromises the expression of essential genes, like *STMN2*, by reducing stability and translation efficiency.

Collectively, these findings underscore a model in which the normal RNA-dependent interactions and phase separation behaviours of TDP-43 become dysregulated by mutations, extrinsic stressors, and alterations in RNA homeostasis. The progressive sequestration of TDP-43 to cytoplasmic aggregates leads to nuclear loss of RNA processing and toxic gain of cytoplasmic function that together contribute to neurodegeneration in ALS [6,19,21,34,50,56,86]. A comprehensive understanding of these interconnected processes is crucial for identifying effective therapeutic strategies aimed at preventing or reversing the noxious assembly of TDP-43.

## TDP-43 as a biomarker in ALS

### Biofluids

The growing body of literature on TDP-43 in ALS has spurred considerable interest in its potential as a biomarker in accessible biofluids. A meta-analysis [87] has provided compelling evidence that cerebrospinal fluid (CSF) levels of TDP-43 are significantly elevated in patients with ALS, reporting an effect estimate of 0.64, albeit with moderate heterogeneity. Furthermore, focusing on TDP-43 in extracellular vesicles (EVs) within the blood may serve as a more reliable surrogate marker for central nervous system pathology. For example, plasma EV-associated TDP-43 levels are markedly elevated in ALS patients compared with healthy controls and other neurodegenerative conditions, with diagnostic performance exceeding that of traditional plasma measurements [88]. These findings highlight the potential of biofluid measures of TDP-43 in ALS, which, in combination with clinical assessment, may improve confidence of diagnostic accuracy and streamline the exclusion of other neuromuscular disorders that ‘mimic’ the clinical presentation of ALS [89]. However, they also underscore the challenges introduced by inter-study variability, stemming from differences in assay methodologies, control populations, and sample processing protocols that currently preclude widespread validation and implementation.

Notably, TDP-43 proteinopathy is also a pathological hallmark of FTD in 50% of cases [58], which is clinically characterised by cognitive symptoms [90], as opposed to motor function deficits and muscle atrophy in ALS [91]. Studies have reported differences in morphology [92] and folding patterns [93] between the cytoplasmic inclusions found in ALS and FTD subtypes that may inform improved specificity for detection of pathological TDP-43 in ALS, but further work is needed to fully characterise the biochemical signatures of TDP-43 pathology in ALS.

Moreover, a review article [94] has detailed the biochemical complexity of TDP-43, noting that its various post-translational modifications and cleavage events render its detection in biofluids particularly challenging. Most assays, such as those employing ELISA, tend to focus on the full-length 45-kDa form, potentially overlooking the disease-specific truncated or phosphorylated isoforms that might more accurately reflect pathological status. This technical limitation is compounded by the interference of CSF proteins like albumin and immunoglobulins that may become more abundant due to blood-brain barrier breakdown in disease, leading to calls for more refined detection methods. Additionally, many antibodies used in current assays are not sufficiently selective for disease-specific forms of TDP-43, often detecting both physiological and pathological species. This lack of specificity could mask the subtle biochemical changes that provide early diagnostic clues.

Emerging technologies, including analysis of TDP-43 misfolding using immuno-infrared sensors [95] and aptamer-based platforms [96,97], offer promising avenues for overcoming these challenges by providing a more detailed analysis of the conformational states, misfolding, and aggregation patterns of TDP-43 in ALS. These platforms also offer higher sensitivity and specificity than traditional immunoassays. A pilot study employing immuno-infrared technology demonstrated that enhanced β-sheet content in CSF could distinguish ALS patients from Parkinson's disease patients and controls and was linked to faster disease progression [95]. However, widespread validation and adoption could be hindered by the requirement of specialist equipment and complex data analysis. Similarly, RNA aptamers have been able to detect TDP-43 oligomers at the nanometre scale using super-resolution microscopy, demonstrating potential for use in early detection and monitoring of TDP-43 aggregates in ALS [97]. However, to date, RNA aptamers have not been used for detection of TDP-43 extracted from biomarker-relevant tissues, such as blood or CSF, only in *ex vivo* brain slices or *in vitro*.

### Peripheral tissues

In a tissue biomarker approach, a skin biopsy study [98] has revealed increased cytoplasmic TDP-43 in dermal fibroblasts of ALS patients. Although these peripheral assessments are still in early stages, they raise the intriguing possibility of using minimally invasive skin biopsies as an additional diagnostic tool, especially when coupled with longitudinal assessments to track disease progression. Additional investigations have explored TDP-43 pathology in peripheral tissues such as the gastrointestinal tract [99] and skeletal muscle [100]. These studies reinforce the concept that TDP-43 pathology extends beyond the central nervous system. However, the variability in tissue sampling, along with the often-small cohorts, underscores the need for larger, methodologically standardised studies before these approaches can be translated into clinical practice.

### Indirect functional assessment

Another promising alternative approach is to infer TDP-43 loss of nuclear function indirectly by measuring downstream cryptic peptides in CSF. This has been achieved using specific monoclonal antibodies designed against a single TDP-43-dependent cryptic peptide [101] as well as mass spectrometry-based detection of a panel of 18 novel cryptic peptides [102]. In addition to serving as a diagnostic biomarker, measuring cryptic peptide levels could also be used in the future as response biomarkers for therapeutic methods aiming to restore TDP-43 nuclear function.

In summary, while impaired TDP-43 status in biofluids and solid tissue is associated with ALS pathology, methodological variability, specificity to ALS, and technical limitations currently preclude the establishment of TDP-43 as a standalone biomarker. Future research should focus on standardising detection methods, improving assay specificity for pathological isoforms, and integrating TDP-43 with other complementary biomarkers to enhance early diagnosis, monitor disease progression, and infer nuclear TDP-43 function. Moreover, combining TDP-43 measurements with other biomarkers, such as neurofilament light chain and tau species, appears to enhance diagnostic accuracy [103], suggesting that a multiplexed biomarker panel might ultimately be the most effective approach.

## TDP-43 as a therapeutic target for ALS

### Approaches targeting nuclear depletion

Preventing or reversing TDP-43 cytoplasmic mislocalisation is a promising therapeutic strategy. Nuclear export inhibitors targeting exportin-1, such as KPT-335 and KPT-350, have been employed to prevent TDP-43 cytoplasmic mislocalisation, modestly improving cell survival and motor performance in preclinical models without significantly elevating nuclear TDP-43 levels [104,105]. The therapeutic potential of synthetic GU-rich sequences has also been explored in preclinical models. Research has demonstrated that multivalent GU oligonucleotides bind multiple TDP-43 molecules, producing high molecular weight complexes that are too large to escape the nuclear pore by passive diffusion [106]. Synthetic GU [106] oligomers were also sufficient to reverse induced cytoplasmic aggregation of TDP-43 but failed to repress cryptic exon inclusion. Thus, a critical challenge of targeting TDP-43 proteinopathy is balancing nuclear localisation with functional preservation, though the authors note the AC-interspersed motif, Clip34nt, as a promising target for enhancing nuclear localisation while preserving the endogenous splicing function of TDP-43.

### Approaches targeting cytoplasmic aggregates

Early prevention of TDP-43 aggregation could also theoretically slow down or stop the progression of disease. A recently published preprint presents a machine learning framework to identify small molecule inhibitors to prevent the formation of TDP-43 aggregates [107]. Of the screening molecules, two identified compounds berberrubine and PE859 were able to decrease TDP-43 aggregation in HEK cells and improve motor deficits in *Caenorhabditis elegans*, underscoring the potential of artificial intelligence to streamline identification of novel drugs.

Enhancing the clearance of misfolded TDP-43 represents another promising avenue. Cytoplasmic TDP-43 aggregates are normally cleared via the UPS and the autophagy-lysosomal pathway. Pharmacological agents that activate these pathways, such as rapamycin, have been used to stimulate autophagy by inhibiting mTOR, thereby reducing the accumulation of toxic TDP-43 C-terminal fragments in cell culture and improving motor function in animal models [108–113]. Rapamycin was well-tolerated in phase II clinical trials but failed to significantly increase regulatory T-cell levels in ALS patients [114]. Alternative autophagy activators, including berberine and trehalose, which act via mTOR-independent activation of TFEB to enhance lysosome biogenesis, have also shown the capacity to reduce TDP-43 aggregation [115–118]. Strategies to augment UPS activity, including the use of deubiquitinating enzyme inhibitors like IU1 and emerging proteolysis-targeting chimeras, are under exploration as well (reviewed in [119]). Although these approaches remain in preclinical stages.

Another major therapeutic strategy in ALS has been to inhibit the aberrant post-translational modifications that characterise TDP-43 aggregates. For example, hyperphosphorylation at serines 409 and 410. Several kinases, including casein kinase 1 (CK-1), tau/tubulin kinases (TTBK1/2), cell division cycle 7 (CDC7), and glycogen synthase kinase 3β (GSK-3β), have been implicated in these modifications [120–124]. Inhibitors of these kinases have shown promise in preclinical studies. Benzothiazole derivatives that selectively inhibit CK-1δ, with nanomolar potency, have reduced TDP-43 phosphorylation in cellular models and extended lifespan in *Drosophila* models [125]. Similarly, TTBK1 inhibitors based on a pyrrolopyrimidine scaffold have demonstrated efficacy in both cellular assays and TDP-43-transgenic mice by restoring the nuclear–cytosolic distribution of TDP-43 [126]. Although CDC7 inhibitors like PHA767491 can reduce phosphorylation levels, their inability to cross the blood–brain barrier limits their clinical utility. The GSK-3β inhibitor Tideglusib reduced TDP-43 phosphorylation in a Prp-hTDP-43A315T mouse model as well as recovered TDP-43 nuclear localisation in human ALS lymphoblasts and a neuroblastoma model [127]. Tidesglusib is planned to enter phase II clinical trials in ALS [128] and has already demonstrated acceptable levels of safety and tolerance in Alzheimer's disease patients, although no clinical benefits were found [129].

It is important for strategies to balance clearance of TDP-43 aggregates with preservation of endogenous function. Monoclonal antibodies that selectively target the C-terminal domain of TDP-43 have been developed to engage Fc gamma receptor–mediated clearance by microglia. This approach not only reduced the burden of toxic TDP-43 species but also preserved the essential physiological functions of the protein in transgenic mouse models [130]. Additionally, small molecule screening and *in silico* docking studies have broadened the therapeutic landscape by identifying compounds that directly bind to only toxic forms of TDP-43. Using the crystal structure of the N-terminal domain of TDP-43, researchers identified nTRD22, a compound that mitigates motor deficits in *Drosophila* models through allosteric modulation, thereby illustrating the potential of structure-based drug design to yield molecules that specifically target pathogenic TDP-43 without interfering with its normal functions [131].

### Genetic approaches

Alternatively, genetic approaches offer means to modulate TDP-43 levels. Work in ENA-modified gapmer antisense oligonucleotides (ASOs) targeting the *TARDBP* transcript has demonstrated that a single intracerebroventricular injection in TDP-43 transgenic mice can produce sustained behavioural improvements and reduce cytoplasmic mislocalisation, even after TDP-43 levels eventually return to baseline [132]. Similarly, nanovector-based delivery of *TARDBP*-targeted siRNA has achieved effective knockdown of TDP-43 *in vitro* while promoting the disassembly of SGs that contain TDP-43 [133]. Conversely, rescuing TDP-43 loss of function in ALS through global expression of TDP-43 risks interfering in the homeostasis of TDP-43 in unaffected cells and potentially worsening progression of disease. To mediate this, recent work used a construct encoding TDP-43 fused to a less aggregation-prone splicing repressor domain (RAVER1), dependent on the expression of an internal TDP-43 sensitive cryptic exon, and was expressed only in cells with knockdown of endogenous TDP-43 [134], rescuing key cryptic splicing events, such as in UNC13A and AARS1. However, the study did not investigate to what extent endogenous cytosolic aggregated TDP-43 may interfere with the nuclear localisation of the TDP-43-RAVER1 fusion protein and therefore its rescue function.

Restoring downstream splicing defects directly has also gained traction as a method of mediating decreased nuclear TDP-43 function, with ASOs to correct cryptic exon splicing in both STMN2 and UNC13A progressing into clinical trials [135,136]. A recent preprint has developed a gene therapy leveraging the small nuclear RNA, U7smOPT, to simultaneously target the mis-splicing of both STMN2 and UNC13A, delivered as a single dose [137]. In iPSC-motor neurons, the vector was able to rescue STMN2 and UNC13A pre-mRNA processing defects, restore STMN2 protein levels, and suppress cryptic exons. Intracerebroventricular delivery of an adeno-associated virus (AAV) vector to a transgenic mouse expressing *STMN2* cryptic exons was also able to restore cortical levels of STMN2 mRNA. These findings highlight the potential of a dual-target AAV-based gene therapy in mediating the RNA dysregulation caused by depleted nuclear TDP-43.

Despite encouraging preclinical findings across multiple approaches, translation to effective clinical therapies has been challenging. While compounds such as kinase inhibitors and ASOs show considerable promise, a critical challenge that remains is achieving a balance between eliminating toxic aggregates and rescuing the essential functions of TDP-43. Moreover, many preclinical studies have relied on genetic models that may not fully recapitulate the subtle and multifactorial nature of ALS. While simpler organisms such as *C. elegans*, Drosophila, and zebrafish show promise in allowing for higher-throughput screening of small molecules, non-genetic animal and iPSC models are needed to better understand the sporadic form of ALS (reviewed in [138]). Future research must refine preclinical models to design interventions that are translatable to the clinical setting and do not compromise the normal biology of TDP-43.

## Future directions—models of ALS

A challenge for studying TDP-43 proteinopathy is the lack of preclinical models that sufficiently capture the complex nature of ALS. To date, research has been limited by conventional TDP-43 transgenic mouse models seldom displaying overt cytoplasmic mislocalisation in spinal motor neurons. This is likely due to many existing models causing transgenic and endogenous expression of TDP-43, an overexpression that causes motor neuron death before the development of cytoplasmic aggregates and the loss of nuclear function (reviewed in [139]). Recent work is increasingly utilising patient-derived iPSC models to study TDP-43 pathology in ALS. A recent preprint generated patient-derived iPSC forebrain organoids harbouring the TDP-43 K181E mutation that recapitulated the cytoplasmic TDP-43 accumulation, RNA dysregulation, and cryptic exon inclusion seen in ALS [140]. Additionally, iPSC-derived neurons have successfully modelled pathological stressors such as chronic oxidative stress [141] and exhibit gene expression and splicing alterations consistent with matched post-mortem tissue [142]. Thus, patient-derived iPSC systems can provide an *in vitro* model for studying TDP-43 pathology that captures complex human physiology and genetics. Such models could advance understanding of the biochemical mechanisms of TDP-43 pathology and improve screening and selection of novel therapies that are most likely to show clinical benefit in ALS patients.

Moreover, a recent investigation revealed that introducing a mutant dynein allele alongside wildtype human TDP-43 led to TDP-43 aggregation and disrupted axonal transport [143]. Another study using a novel mouse model that combines the Loa (F580Y) dynein cytoplasmic 1 heavy chain 1 (DYNC1H1) mutation with a cholinergic neuron-specific *Dync1h1* knockout demonstrates that disrupting axonal transport alone is sufficient to trigger early TDP-43 aggregation, highlighting that TDP-43 pathology relevant to ALS can be induced without directly manipulating TDP-43 expression, and thus, specialised TDP-43 mouse models need not exclusively rely on direct manipulation of TDP-43 [144].

Future work should also aim to investigate the impacts of TDP-43 proteinopathy in non-neuronal cell types and possible non-cell autonomous mechanisms. For example, work using a humanised TDP-43 M337V transgenic mouse model revealed that aberrant TDP-43 function in microglia leads to significant dysregulation of microglia-derived miRNAs critical for neuronal development and function [51]. A potential hypothesis is that microglia-derived, miRNA-containing vesicles are endocytosed by neighbouring neurons, where they alter gene transcription [145], outlining a role for neuroinflammatory processes in ALS pathology [51]. While the causes and consequences of TDP-43-induced microglial dysfunction remain incompletely understood, these findings emphasise the importance of a cell-type-specific approach to understanding pathological heterogeneity.

## Conclusion

The role of TDP-43 in ALS as a major disease protein has been known for over two decades. The causes and impacts of the cytoplasmic aggregation and mislocalisation of TDP-43 are multifactorial, which leads to complexity in measuring TDP-43 levels as a biomarker and in developing therapeutics targeting TDP-43 pathology. Further studies, using relevant models, are required to better understand TDP-43 pathology in ALS.

## Perspectives

- The RNA-binding protein TDP-43 is known as a major disease protein in ALS, a devastating disease that currently has no cure.
- The past two decades of research have shown TDP-43 pathology in ALS to be characterised by mislocalisation from nucleus to cytoplasm, highlighting both toxic aggregation and loss of physiological function as key drivers of neurodegeneration. Leading on from this research, TDP-43 shows promise as a biomarker and therapeutic target in ALS.
- Future directions should include the use of pre-clinical models that better recapitulate TDP-43 pathology, standardising detection methods for biomarker development, and further research into treatment strategies that balance addressing TDP-43 toxicity whilst retaining normal TDP-43 function.

## Open Access

Open access for this article was enabled by the participation of the University of Sussex in an all-inclusive Read & Publish agreement with Portland Press and the Biochemical Society under an agreement with JISC.

## Abbreviations

AAV: adeno-associated virus
ALS: amyotrophic lateral sclerosis
ASOs: antisense oligonucleotides
CK-1: casein kinase 1
CRISPR: clustered regularly interspaced short palindromic repeats
CSF: cerebrospinal fluid
CTD: C-terminal domain
DYNC1H1: dynein cytoplasmic 1 heavy chain 1
EVs: extracellular vesicles
FTD: frontotemporal dementia
GSK-3β: glycogen synthase kinase 3β
iPSCs: induced pluripotent stem cells
LLPS: liquid–liquid phase separation
NMD: nonsense-mediated decay
NTD: N-terminal domain
SG: stress granule
STMN2: stathmin 2
TDP-43: TAR DNA-binding protein 43
UTR: untranslated region
UPS: ubiquitin-proteasome system
